# Applications of Traditional and Digital Anthropometric Measures in Orthopaedics and Rehabilitation: A Systematic Review

**DOI:** 10.7759/cureus.116512

**Published:** 2026-09-18

**Authors:** Humera A Bagwan, Sandeep Shinde

**Affiliations:** 1 Department of Musculoskeletal Sciences, Krishna College of Physiotherapy, Krishna Vishwa Vidyapeeth (Deemed To Be University), Karad, IND

**Keywords:** anthropometric factors, anthropometry, body composition, clinical assessment, digital anthropometry, measuring tools, orthopaedic diseases, orthopaedics, rehabilitation, rehabilitation outcomes.

## Abstract

Anthropometry offers noninvasive measurements of body size, proportions, and composition that can inform musculoskeletal assessment and rehabilitation. Traditional anthropometric measures continue to be widely used owing to their accessibility, whereas digital technologies provide further approaches for objective body and movement assessment. However, evidence on the applications and measurement properties of traditional and digital anthropometric measures across orthopaedic and rehabilitation settings remains inconsistent. This systematic review assessed the measurement methods, clinical applications, and reported implications of traditional and digital anthropometric measures in orthopaedic assessment and rehabilitation.

This systematic review was performed in accordance with the Preferred Reporting Items for Systematic Reviews and Meta-Analyses (PRISMA) 2020 Statement. We searched PubMed/MEDLINE, Scopus, Web of Science, Cochrane Library, PEDro, and Google Scholar for studies published between January 2017 and July 2026, with the final search performed in July 2026. English-language full-text studies involving human participants that evaluated anthropometric measures in orthopaedic, musculoskeletal, or rehabilitation settings were included. Cross-sectional, observational, cohort, randomized controlled, diagnostic, validation, and reliability studies were eligible for inclusion. Risk of bias and methodological quality were evaluated using tools appropriate to each study design, including Consensus-Based Standards for the Selection of Health Measurement Instruments (COSMIN) for measurement-validation studies, Quality Assessment of Diagnostic Accuracy Studies-2 (QUADAS-2 ​​​​​​) for diagnostic-accuracy studies, the Cochrane Risk of Bias Assessment Tool 2 (RoB 2) for randomized controlled trials, and the Joanna Briggs Institute’s (JBI) Critical Appraisal Checklist for analytical cross-sectional studies. Owing to substantial clinical and methodological heterogeneity, the findings were synthesized narratively.

Eighteen studies met the inclusion criteria: six diagnostic/validation studies, eight analytical cross-sectional studies, and four randomized controlled trials. Traditional and digital anthropometric methods were applied for body measurement, screening, risk stratification, preoperative planning, and rehabilitation assessment and monitoring. Validation studies generally reported good agreement or reliability, whereas cross-sectional studies identified associations with disease severity, bone health, low back pain, graft dimensions, and postural characteristics. Randomized controlled trials assessed anthropometric and related measures in rehabilitation for low back pain, knee osteoarthritis, and total knee arthroplasty. The risk-of-bias assessment indicated a generally low risk, although two randomized controlled trials raised some concerns regarding selection of the reported result.

Traditional and digital anthropometric measures can complement orthopaedic assessment and rehabilitation planning when selected, measured, and interpreted according to standardized protocols. However, heterogeneity across studies and limited evidence of their direct effects on patient outcomes highlight the need for further standardized, prospective research.

## Introduction and background

The scientific study of the dimensions, proportions, form, and composition of the human body is called anthropometry. The term derives from the Greek words anthropos (human) and metron (measure). Anthropometric assessment provides quantitative information regarding body size and body composition and typically includes measurements such as stature, body mass, BMI, limb length, circumferences, breadths, skinfold thickness, and body-segment proportions [1]. These measurements are generally simple to perform, noninvasive, relatively inexpensive, and suitable for repeated assessment. Anthropometry has therefore been used in healthcare to characterize body size and composition, nutritional status, growth, and musculoskeletal characteristics [2,3].

Historically, anthropometry was used mainly to describe human growth, body proportions, and population characteristics. Its clinical application has expanded alongside developments in anatomy, biomechanics, rehabilitation science, and standardized measurement procedures [1,4]. Body dimensions, proportions, and composition may be associated with clinically relevant characteristics such as posture, mobility, physical performance, and joint loading; these relationships, however, are influenced by the population studied, the measurement protocol, and the clinical context [1,4]. Anthropometric measurements should therefore be interpreted as quantitative characteristics of the body and not as direct measures of function or treatment effectiveness.

In orthopaedic practice, anthropometric assessment may complement clinical evaluation by providing information on body dimensions, limb lengths, circumferences, proportions, and body composition. This information has potential relevance to patient characterization, assessment of musculoskeletal characteristics, screening, preoperative and surgical planning, implant or component selection, and postoperative monitoring [3,4]. Anthropometric variables may also be examined for their relationship to clinical characteristics such as pain, mobility, gait, or functional limitation. However, an association between an anthropometric characteristic and a clinical outcome does not in itself demonstrate that anthropometric assessment improves diagnosis, treatment selection, surgical outcomes, or recovery.

Anthropometry may also be used in rehabilitation to monitor changes in body mass, limb circumference, muscle size, edema, and body composition over the course of care [3-5]. Repeated measurements can provide information about changes in selected physical characteristics and may complement functional and clinical outcome assessments. Nonetheless, the presence of anthropometric measurements within a rehabilitation study does not necessarily indicate that anthropometry was itself the intervention or that anthropometric assessment caused an improvement in rehabilitation outcomes on its own. Its clinical value depends on the specific measurement, assessment protocol, population, and intended use.

It is also important to distinguish anthropometric measurement from related clinical, biomechanical, and physiological assessments. Goniometric range-of-motion measurements, gait and spatiotemporal parameters, inertial-sensor kinematics, motion-capture variables, force-platform outputs, center-of-pressure or posturographic measures, joint moments, imaging findings, limb occlusion pressure, and blood pressure are not considered anthropometric measures. They may, however, be evaluated alongside anthropometric variables as associated clinical, functional, biomechanical, or physiological outcomes. Likewise, body-composition techniques may be considered within an anthropometric framework when they quantify characteristics such as body mass, fat mass, lean mass, muscle mass, or body-fat percentage.

Traditional anthropometric methods include tape measures, stadiometers, weighing scales, calipers, and anthropometers. Standardized measurement procedures, appropriate equipment, and quality-control practices are important for minimizing measurement error and improving consistency in anthropometric assessment [6]. These methods remain applicable because of their practicality and portability in clinical settings. Advances in technology have also introduced digital approaches, including three-dimensional body scanning, wearable sensors, smartphone-based systems, and artificial-intelligence-assisted measurement [7]. Digital anthropometry may facilitate rapid and standardized data acquisition; measurement accuracy, reliability, and clinical usefulness, however, may vary depending on the technology, protocol, population, and intended application.

Anthropometric measures have been studied across a range of orthopaedic and rehabilitation contexts. Depending on the clinical context, anthropometry has been used for body-composition assessment, structural characterization, screening, surgical planning, and rehabilitation monitoring. The diversity of these applications reflects the potential breadth of anthropometry but also introduces substantial variation in populations, measurement techniques, clinical purposes, and outcomes.

Previous studies and systematic reviews have examined specific anthropometric techniques, particularly the precision, reliability, and accuracy of digital anthropometric systems [8,9]. However, evidence concerning anthropometric measurement properties does not necessarily address how these measures are applied across broader orthopaedic and rehabilitation settings or whether their use is associated with clinically meaningful patient outcomes. Existing literature includes observational studies, cross-sectional associations, measurement-validation studies, diagnostic or screening investigations, and clinical trials in which anthropometry may serve as an assessment variable rather than an intervention. This heterogeneity makes it important to distinguish measurement validity and reliability from clinical application and from evidence of improved patient outcomes.

Unlike previous reviews that have primarily focused on the measurement properties of digital anthropometric technologies, the present review examines the broader clinical application of both traditional and digital anthropometric measures across orthopaedic and musculoskeletal rehabilitation settings, including clinical characterization, screening, surgical planning, body-composition assessment, and rehabilitation monitoring.

A comprehensive synthesis is therefore warranted to clarify how traditional and digital anthropometric measures are used across orthopaedic and musculoskeletal rehabilitation settings, what measurement methods and clinical purposes are reported, and what evidence supports their validity, reliability, diagnostic or clinical utility, and association with patient-related outcomes. Accordingly, this systematic review aimed to describe the clinical applications, measurement methods, and reported validity, reliability, diagnostic utility, or prognostic relevance of traditional and digital anthropometric measures in orthopaedic and musculoskeletal rehabilitation settings. The review examined their use in clinical characterization, body-composition assessment, screening, surgical planning, and rehabilitation monitoring, while distinguishing measurement validity and clinical associations from evidence of improved patient outcomes.

Figure 1 presents the conceptual framework used in this review to show the potential clinical applications of anthropometry in orthopaedic and rehabilitation settings. The framework distinguishes direct anthropometric measurements from associated clinical, functional, biomechanical, and physiological outcomes.

**Figure 1:**
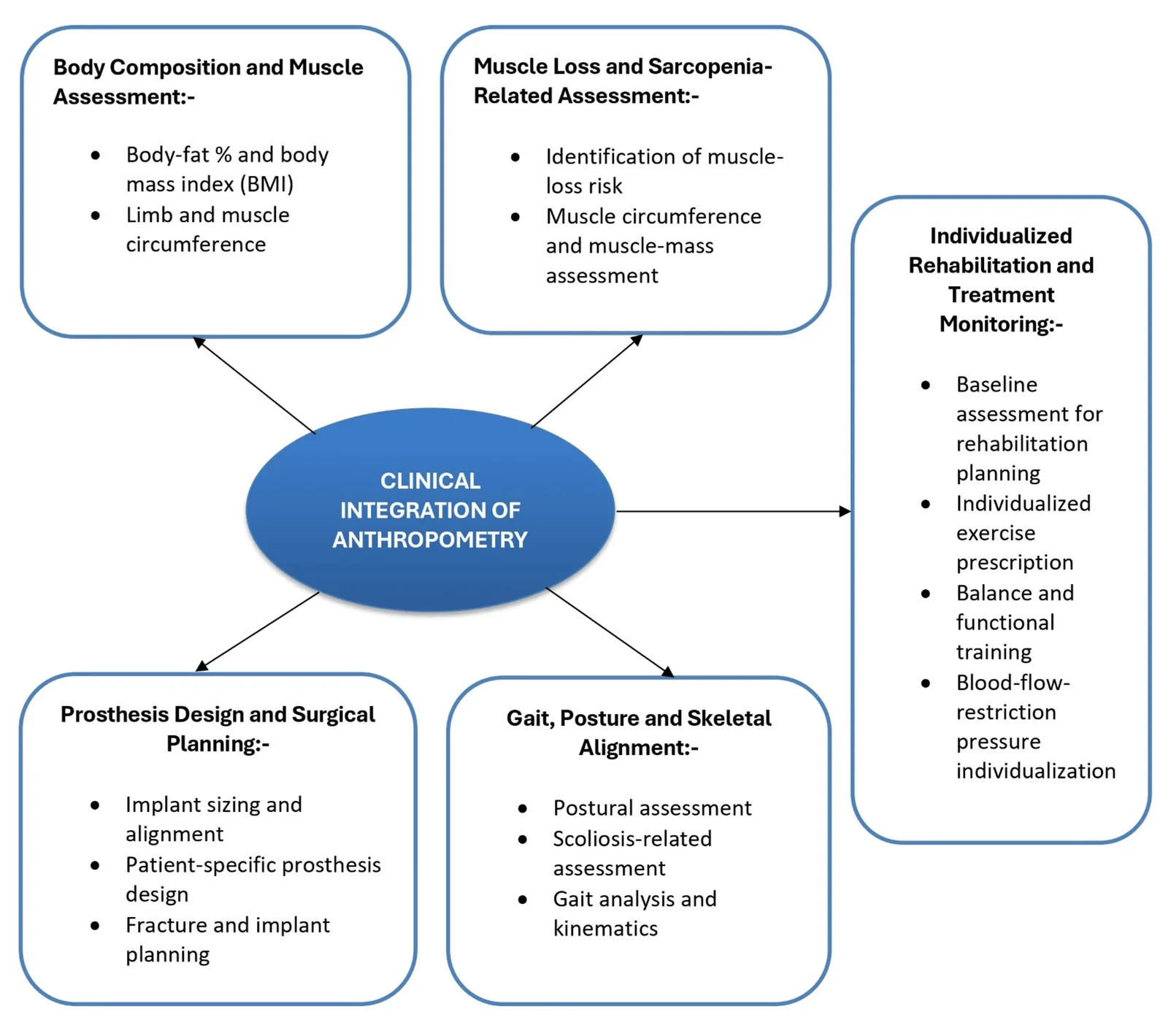
Conceptual framework of the clinical integration of anthropometry in orthopaedic and evidence-based rehabilitation

## Review

Methodology

Study Design

This systematic review was conducted following the Preferred Reporting Items for Systematic Reviews and Meta-Analyses (PRISMA) 2020 Statement [10]. The review was designed to synthesize evidence regarding the applications and measurement characteristics of traditional and digital anthropometric measures in orthopaedic assessment and rehabilitation. The review addressed three prespecified evidence domains: (1) measurement characteristics, including validity and reliability; (2) associations between anthropometric measures and orthopaedic or musculoskeletal characteristics; and (3) reported applications in orthopaedic assessment, surgical planning, rehabilitation assessment, and monitoring. The eligibility criteria, outcomes of interest, evidence domains, and narrative synthesis approach were all specified before study screening. The review protocol was not prospectively registered with PROSPERO or the Open Science Framework, which may limit the availability of an independently accessible record of the prespecified methodology.

Search Strategy

A comprehensive electronic literature search was conducted in PubMed/MEDLINE, Scopus, Web of Science, PEDro, the Cochrane Library, and Google Scholar to identify studies investigating the applications, measurement characteristics, and clinical use of traditional and digital anthropometric measures in orthopaedic, musculoskeletal, and rehabilitation settings. The search covered studies published from January 2017 to July 2026, with the final search conducted in July 2026.

The search strategy combined controlled vocabulary, where available, with database-specific free-text terms related to anthropometry, digital anthropometry, body dimensions, body composition, BMI, limb dimensions, circumferences, muscle mass, skinfold thickness, orthopaedic conditions, musculoskeletal conditions, rehabilitation, physiotherapy, and physical therapy. Boolean operators (AND and OR) were used to combine the search concepts, and database-specific indexing terms, search fields, and syntax were adapted to the requirements of each information source.

The complete PubMed/MEDLINE search strategy was as follows:

("Anthropometry"[Mesh] OR anthropometr[Title/Abstract] OR "anthropometric assessment"[Title/Abstract] OR "digital anthropometry"[Title/Abstract] OR "body dimensions"[Title/Abstract] OR "body composition"[Title/Abstract] OR "body mass index"[Title/Abstract] OR BMI[Title/Abstract] OR "body circumference"[Title/Abstract] OR "limb length"[Title/Abstract] OR "limb circumference"[Title/Abstract] OR "muscle mass"[Title/Abstract] OR "skinfold thickness"[Title/Abstract]) AND (orthopedic[Title/Abstract] OR orthopaedic*[Title/Abstract] OR musculoskeletal[Title/Abstract] OR rehabilitation[Title/Abstract] OR physiotherapy[Title/Abstract] OR "physical therapy"[Title/Abstract] OR "clinical assessment"[Title/Abstract] OR "orthopaedic assessment"[Title/Abstract]))**

The complete database-specific search strategies for Scopus, Web of Science, PEDro, the Cochrane Library, and Google Scholar, together with the corresponding numbers of records retrieved, are presented in Table 1.

**Table 1:** Database-specific search strategies and records retrieved AND: Boolean operator used to combine search concepts; OR: Boolean operator used to broaden the search by including alternative terms; PEDro: Physiotherapy Evidence Database; MEDLINE: Medical Literature Analysis and Retrieval System Online; TITLE-ABS-KEY: title, abstract, and keyword fields in Scopus; TS: Topic search field in Web of Science; MeSH: Medical Subject Headings; BMI: body mass index

Database/information source	Search strategy (Boolean search string)	Records retrieved (n)
PubMed/MEDLINE	("Anthropometry"[Mesh] OR anthropometr*[Title/Abstract] OR "anthropometric assessment"[Title/Abstract] OR "digital anthropometry"[Title/Abstract] OR "body dimensions"[Title/Abstract] OR "body composition"[Title/Abstract] OR "body mass index"[Title/Abstract] OR BMI[Title/Abstract] OR "body circumference"[Title/Abstract] OR "limb length"[Title/Abstract] OR "limb circumference"[Title/Abstract] OR "muscle mass"[Title/Abstract] OR "skinfold thickness"[Title/Abstract]) AND (orthopedic*[Title/Abstract] OR orthopaedic*[Title/Abstract] OR musculoskeletal[Title/Abstract] OR rehabilitation[Title/Abstract] OR physiotherapy[Title/Abstract] OR "physical therapy"[Title/Abstract] OR "clinical assessment"[Title/Abstract] OR "orthopaedic assessment"[Title/Abstract])	138
Scopus	TITLE-ABS-KEY(anthropometr* OR "anthropometric assessment" OR "digital anthropometry" OR "body dimensions" OR "body composition" OR "body mass index" OR BMI OR "body circumference" OR "limb length" OR "limb circumference" OR "muscle mass" OR "skinfold thickness") AND TITLE-ABS-KEY(orthopedic* OR orthopaedic* OR musculoskeletal OR rehabilitation OR physiotherapy OR "physical therapy" OR "clinical assessment" OR "orthopaedic assessment")	104
Web of Science	TS=(anthropometr* OR "anthropometric assessment" OR "digital anthropometry" OR "body dimensions" OR "body composition" OR "body mass index" OR BMI OR "body circumference" OR "limb length" OR "limb circumference" OR "muscle mass" OR "skinfold thickness") AND TS=(orthopedic* OR orthopaedic* OR musculoskeletal OR rehabilitation OR physiotherapy OR "physical therapy" OR "clinical assessment" OR "orthopaedic assessment")	76
PEDro	(anthropometry OR anthropometric OR "digital anthropometry" OR "body mass index" OR "body composition" OR "limb length" OR circumference) AND (orthopaedic OR orthopedic OR musculoskeletal OR rehabilitation OR physiotherapy OR "physical therapy")	34
Cochrane Library	("anthropometry" OR anthropometric OR "digital anthropometry" OR "body dimensions" OR "body composition" OR "body mass index" OR BMI OR "body circumference" OR "limb length" OR "limb circumference" OR "muscle mass" OR "skinfold thickness") AND (orthopaedic OR orthopedic OR musculoskeletal OR rehabilitation OR physiotherapy OR "physical therapy" OR "clinical assessment")	26
Google Scholar	("anthropometry" OR "anthropometric assessment" OR "digital anthropometry" OR "body composition" OR "body mass index" OR "limb length" OR "limb circumference") AND (orthopaedic OR orthopedic OR musculoskeletal OR rehabilitation OR physiotherapy OR "physical therapy")	42
Total		420

The following limits were applied where applicable: publication dates from January 2017 to July 2026, and English language. The publication-date restriction was applied to focus the review on contemporary evidence relevant to orthopaedic and rehabilitation practice. The English-language restriction was part of the predefined eligibility criteria. Trial registries, theses and dissertations, preprint servers, and other grey-literature sources were not searched.

Google Scholar was searched separately in July 2026, using predefined combinations of anthropometry-related and orthopaedic/rehabilitation-related keywords. The retrieved records were screened in sequence according to the predefined eligibility criteria, with a total of 42 records screened. Reference lists of included studies and relevant review articles were also manually screened to identify further eligible studies. Forward citation searching was not performed. Records identified through backward citation searching were assessed using the same predefined eligibility criteria.

All retrieved records were imported into EndNote, where 82 duplicate records were identified and removed using its duplicate-detection function before screening. The literature searches and initial duplicate removal were carried out by one reviewer and independently verified by a second. A total of 420 records were identified across the six information sources: PubMed/MEDLINE (n = 138), Scopus (n = 104), Web of Science (n = 76), PEDro (n = 34), the Cochrane Library (n = 26), and Google Scholar (n = 42). After removing 82 duplicates, 338 records remained for title and abstract screening.

Selection Criteria

Inclusion criteria: Original full-text research studies published in English that involved human participants of any age or gender were eligible. Studies were included when traditional or digital anthropometric measures formed a substantive component of the investigation and were evaluated within a clearly defined orthopaedic, musculoskeletal, surgical, or rehabilitation context. To be eligible, a study had to address at least one prespecified evidence domain: (1) measurement validity or reliability; (2) diagnostic or prognostic accuracy, where applicable; (3) associations between anthropometric measures and orthopaedic, musculoskeletal, or clinically relevant outcomes; (4) applications in orthopaedic assessment, surgical planning, rehabilitation assessment, or monitoring; or (5) feasibility, usability, or clinical utility of digital anthropometric measurement systems. Anthropometric measures eligible for inclusion included BMI, body composition, limb length, limb circumference, body proportions, muscle mass, skinfold thickness, and other standardized traditional or digital body dimensions. Eligible study designs included observational, cross-sectional, randomized controlled, diagnostic, validation, and reliability studies.

Exclusion criteria: Non-English articles, studies involving animals or cadavers, conference abstracts, editorials, letters, case reports, case series, narrative reviews, systematic reviews, and meta-analyses were all excluded. Studies with inaccessible full text, duplicate publications, overlapping datasets, or insufficient methodological information were likewise excluded. Studies were excluded if anthropometric measures had no clearly defined orthopaedic, musculoskeletal, surgical, or rehabilitation relevance. Also excluded were studies focused exclusively on general obesity assessment, cardiovascular or cardiometabolic risk prediction, body-composition assessment in healthy populations without an orthopaedic or rehabilitation application, BFR pressure prediction without a direct orthopaedic or musculoskeletal rehabilitation application, and general body-scanning or three-dimensional scanning validation without a defined orthopaedic, musculoskeletal, surgical, or rehabilitation application. Studies in which height, weight, or BMI were reported only as baseline or descriptive participant characteristics, without assessment in relation to a prespecified evidence domain, were also excluded.

Table 2 summarizes the eligibility criteria for study selection.

**Table 2:** Eligibility criteria for study selection RCT: randomized controlled trial; BMI: body mass index; BFR: blood flow restriction

Category	Inclusion criteria	Exclusion criteria
Population	Human participants of any age or gender with a defined orthopaedic, musculoskeletal, surgical, or rehabilitation relevance	Animal or cadaveric studies; human studies without relevant orthopaedic, musculoskeletal, surgical, or rehabilitation context
Anthropometric measurement/exposure	Traditional or digital anthropometric measures, including BMI, body composition, limb length, limb circumference, body proportions, muscle mass, skinfold thickness, and standardized digital/3D body dimensions	Anthropometric measures reported only descriptively or without evaluation relevant to the study objective
Clinical context	Orthopaedic, musculoskeletal, surgical, or rehabilitation assessment, surgical planning, rehabilitation assessment, monitoring, or clinically relevant digital measurement application	General obesity, cardiovascular/cardiometabolic risk, healthy-population body composition without orthopaedic/rehabilitation relevance, unrelated BFR pressure prediction, or general body-scanning validation without relevant clinical application
Evidence domain	(1) validity/reliability; (2) diagnostic/prognostic accuracy, where applicable; (3) associations with orthopaedic, musculoskeletal, or clinical outcomes; (4) orthopaedic assessment, surgical planning, rehabilitation assessment, or monitoring; or (5) feasibility, usability, or clinical utility of digital anthropometric systems	Studies not addressing at least one prespecified evidence domain
Comparator	Comparator required where applicable for comparative effectiveness or diagnostic/prognostic studies; not mandatory for validation, reliability, association, or feasibility studies	Comparative studies without an appropriate comparator where a comparator is required to answer the stated research question
Study type	Original full-text research articles, including observational, cross-sectional, RCT, diagnostic, validation, and reliability studies	Conference abstracts, editorials, letters, case reports, case series, narrative reviews, systematic reviews, and meta-analyses
Data availability	Full-text articles with sufficient methodological information and relevant data/outcomes for extraction	Unavailable full text, duplicate publications, overlapping datasets, or insufficient methodological information

Study Selection and Data Extraction

PRISMA 2020 recommendations were followed throughout study selection. Once duplicate records were removed, studies were screened according to the predetermined eligibility criteria. Study selection was carried out independently by two reviewers, with any disagreements resolved through discussion and consensus. The PRISMA 2020 flow diagram was used to document the study-selection process [10].

Data were independently extracted by two reviewers, using a standardized data-extraction form. The extracted information included study title, author, year of publication, study design, participant characteristics, anthropometric parameters, measurement methods or tools, evidence domain, clinical or research purpose, outcome measures, and principal findings. Studies were classified according to the prespecified evidence domains : (1) measurement characteristics, including validity and reliability; (2) clinical associations between anthropometric measures and orthopaedic or musculoskeletal characteristics; and (3) reported clinical applications in orthopaedic assessment, surgical planning, rehabilitation assessment, or monitoring. Data extracted by both reviewers were compared for consistency, and any discrepancies were resolved through discussion and consensus. The final agreed -upon dataset was used for the narrative synthesis.

Quality Assessment

Risk of bias was assessed using appraisal tools suited to the methodological characteristics and objectives of each included study. Two reviewers independently assessed the risk of bias for all included studies using the prespecified assessment tools. Randomized controlled trials were assessed using the Cochrane Risk of Bias 2 (RoB 2) tool [11]; analytical cross-sectional studies were assessed using the Joanna Briggs Institute (JBI) Critical Appraisal Checklist for Analytical Cross-Sectional Studies [12]; studies evaluating measurement properties such as reliability, agreement, or measurement validity were assessed using an appropriate Consensus-Based Standards for the Selection of Health Measurement Instruments (COSMIN) risk-of-bias framework, where applicable; and studies that specifically evaluated diagnostic accuracy were assessed using the Quality Assessment of Diagnostic Accuracy Studies-2 (QUADAS-2) tool [13].

Assessments were performed independently, then compared between the two reviewers. Any disagreements were resolved through discussion and consensus. No formal calibration exercise was performed before the full appraisal. Each judgement was based on information reported in the corresponding primary study, while supporting methodological evidence and reviewer justifications were recorded in the detailed risk-of-bias assessment tables. Risk-of-bias judgements were factored into the narrative synthesis and interpretation of the findings; studies with greater concerns were not treated as equivalent to those with lower risk of bias, and the certainty or interpretation of individual findings was qualified accordingly. Risk-of-bias assessments were reported as domain-level judgements rather than converted into unsupported overall categories such as "high quality."

Data Synthesis

Substantial clinical and methodological heterogeneity across the included studies precluded quantitative pooling; as such, findings were synthesized narratively in line with SWiM guidance [14]. Studies were grouped by primary purpose into five categories: (1) traditional anthropometric measurements, (2) digital body-size and body-shape measurements, (3) surgical planning and implant design, (4) prognostic, correlational, or association-based applications, and (5) rehabilitation assessment and monitoring. Within each category, studies were compared on the basis of population, anthropometric measure, measurement method, clinical context, comparator (where applicable), outcomes, and study design. Where reported, study-level quantitative findings were summarized according to the relevant evidence domain, including reliability and agreement coefficients for measurement-validation studies, diagnostic accuracy estimates for diagnostic studies, correlation or regression estimates for association-based studies, and between-group effects for randomized controlled trials.

Relevant quantitative findings, including reliability/agreement measures, validity estimates, sensitivity, specificity, correlation or regression estimates, between-group effects, and confidence intervals, were extracted and reported wherever available. No additional effect estimates were calculated where data were insufficient or studies were not comparable. As these measures addressed different constructs and were reported across heterogeneous populations and study designs, they were not combined into a common pooled estimate. The direction and magnitude of findings were interpreted within the context of study design, clinical application, and methodological limitations.

Statistical significance was not treated as synonymous with clinical importance; effect magnitude, uncertainty, measurement performance, clinical context, and patient relevance were all considered alongside reported p-values, where available. Risk-of-bias judgements were incorporated into the interpretation, and studies with greater methodological concerns were interpreted with caution. Measurement performance, clinical applications, prognostic/associational evidence, and rehabilitation-related findings were kept distinct rather than being combined into a single overall effect estimate.

Results

PubMed/MEDLINE, Scopus, Web of Science, PEDro, Cochrane Library, and Google Scholar were searched, yielding 420 records. After removal of 82 duplicate records, 338 records remained for title and abstract screening, of which 286 were excluded. Fifty-two reports were sought for retrieval, of which two could not be retrieved. The remaining 50 full-text reports were assessed for eligibility. Of these, 32 reports were excluded for the following reasons: not related to clinical anthropometry (n = 10), non-orthopaedic/non-rehabilitation studies (n = 8), anthropometry reported only as baseline characteristics (n = 6), review articles, conference abstracts, or editorials (n = 5), and insufficient outcome data (n = 3). Eighteen studies met the predefined eligibility criteria and were included in the qualitative synthesis. The PRISMA 2020 flow diagram presents the complete study-selection process (Figure 2).

**Figure 2:**
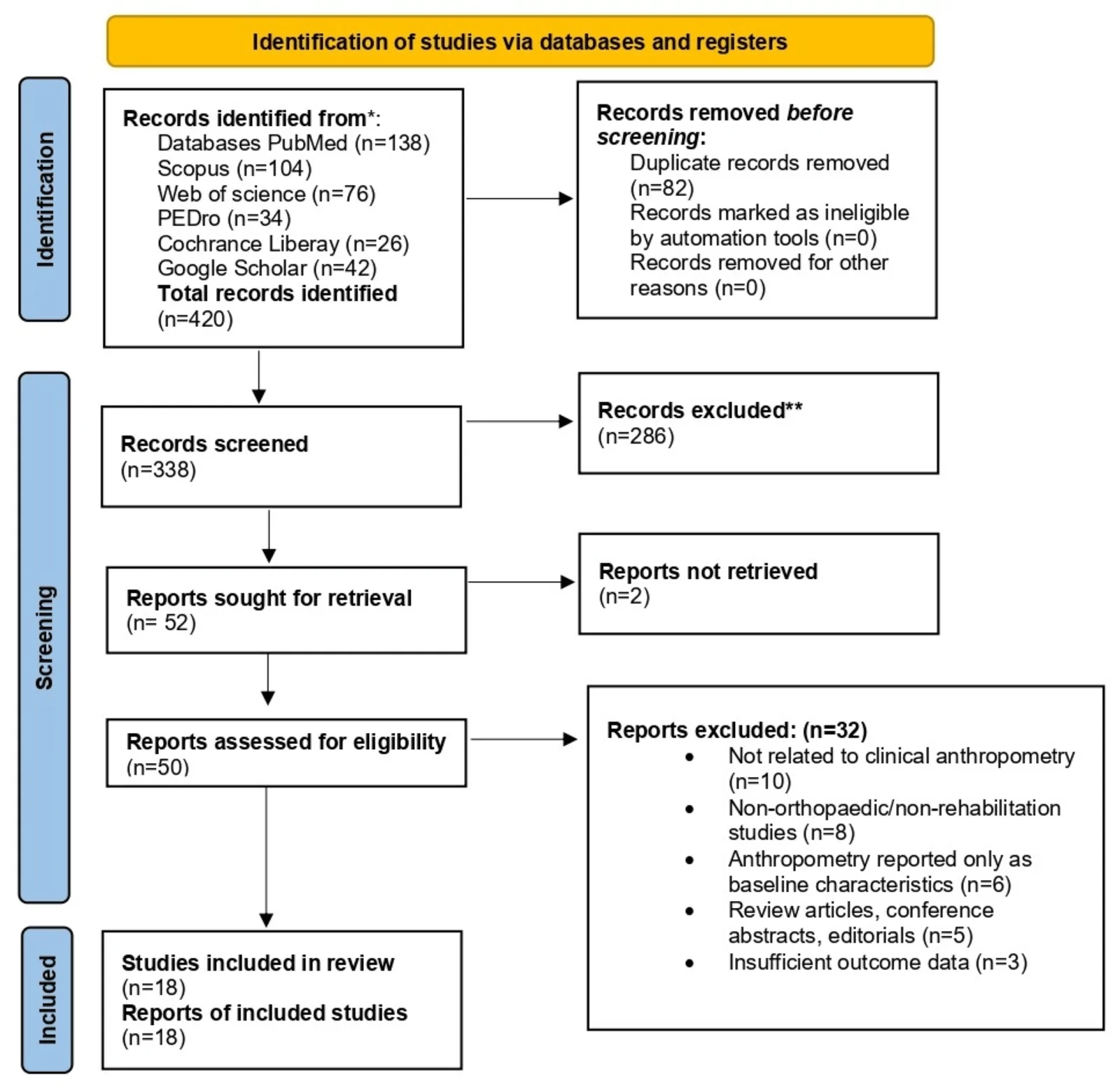
PRISMA flow diagram depicting study selection PRISMA: Preferred Reporting Items for Systematic Reviews and Meta-Analyses

Overall, the 18 included studies demonstrated varied applications of anthropometric assessment across orthopaedic, musculoskeletal, surgical, and rehabilitation settings. Traditional and digital methods were used to assess body size, circumferences, limb dimensions, body composition, and body shape. Applications included measurement validation, musculoskeletal assessment, surgical and implant planning, individualized orthotic and rehabilitation applications, and evaluation of clinical and functional outcomes. Associated measures such as range of motion (ROM), gait, centre of pressure, blood pressure, and limb occlusion pressure were considered clinical or functional outcomes rather than anthropometric measures. Table 3 summarizes the characteristics, anthropometric measures, clinical applications, and main findings of the included studies.

**Table 3:** Characteristics, anthropometric measures, associated clinical measures, and key findings of the included studies Anthropometric measures were classified separately from associated clinical, functional, biomechanical, physiological, and motion-analysis measures. Joint range of motion, center-of-pressure/posturographic variables, gait parameters, inertial-sensor kinematics, limb occlusion pressure, and blood pressure were not classified as anthropometric measures. These variables are reported as associated clinical or outcome measures when evaluated alongside anthropometric characteristics ADP: air displacement plethysmography; ASAS-HI: Assessment of SpondyloArthritis International Society Health Index; ASDAS-CRP: Ankylosing Spondylitis Disease Activity Score using C-reactive protein; BASDAI: Bath Ankylosing Spondylitis Disease Activity Index; BASFI: Bath Ankylosing Spondylitis Functional Index; BASMI: Bath Ankylosing Spondylitis Metrology Index; BASRI: Bath Ankylosing Spondylitis Radiology Index; BFR: blood-flow restriction; BIA: bioelectrical impedance analysis; BMI: body mass index; BMD: bone mineral density; BP: blood pressure; BUA: broadband ultrasound attenuation; CNN: convolutional neural network; COP: center of pressure; CRP: C-reactive protein; CT: computed tomography; CV: coefficient of variation; DXA: dual-energy X-ray absorptiometry; ICC: intraclass correlation coefficient; IMU: inertial measurement unit; KAM: knee adduction moment; KOOS: Knee injury and Osteoarthritis Outcome Score; LMV: local muscle vibration; mSASSS: modified Stoke Ankylosing Spondylitis Spine Score; QUS: quantitative ultrasound; ROM: range of motion; SOS: speed of sound; T-score: bone mineral density T-score; TEM: technical error of measurement; TUG: Timed Up and Go; VAS: visual analogue scale; WOMAC: Western Ontario and McMaster Universities Osteoarthritis Index; WHR: waist-to-hip ratio; WHtR: waist-to-height ratio

No.	Study	Study design	Population/clinical context	Anthropometric measure(s)	Associated clinical/functional measure(s)	Measurement method/instrument	Clinical application	Main quantitative findings
1	Bourgeois et al. (2017) [15]	Cross-sectional study	Clinical anthropometric assessment and body-composition evaluation	Waist, hip, arm, and thigh circumferences; regional and total body volume	Body-composition measures	3D optical scanners (KX-16, Proscanner, Styku), flexible tape, DXA, ADP	Validation and comparison of digital and conventional anthropometric methods for body-size and body-shape assessment	Optical scanners demonstrated good agreement/validity compared with reference methods, supporting their use for clinical anthropometric measurement
2	Bragança et al. (2018) [16]	Comparative validation study	Clinical anthropometric and rehabilitation assessment	Body circumferences and body dimensions	Precision, reliability, TEM, ICC, CV	Measuring tape; Kinect-based 3D body scanner	Comparison of manual and digital anthropometric measurement methods	Manual measurements showed better precision, while both methods demonstrated good reliability
3	Beckmann et al. (2019) [17]	Cross-sectional validation study	Body-size/body-composition assessment in young adults	Height, waist circumference, BMI	Body-fat/body-composition measures	3D laser body scanner, stadiometer, measuring tape, BIA	Validation of 3D digital anthropometric measurements against conventional methods	Excellent agreement was observed between digital and manual measurements, although small systematic differences were present
4	Tinsley et al. (2020) [18]	Cross-sectional validation study	Body-composition assessment	Waist, hip, thigh, neck and arm circumferences; body volume	Body-composition estimates	Four 3D optical scanners, DXA, ADP	Evaluation of digital anthropometric systems for body-size and body-volume assessment	3D scanning demonstrated high precision, although validity varied across measurement systems
5	Chen et al. (2020) [19]	Cross-sectional study	Patients with ankylosing spondylitis	Waist-to-height ratio, waist circumference, BMI	CRP, ESR, ASDAS-CRP, BASMI, BASFI, BASDAI, BASRI, mSASSS, ASAS-HI and blood pressure	Digital weighing scale, stadiometer, measuring tape	Evaluation of anthropometric indices in relation to disease severity and functional/clinical characteristics in a musculoskeletal disorder	Central obesity measures, particularly waist-to-height ratio and waist circumference, were more strongly associated with disease severity than BMI
6	Kennedy et al. (2020) [20]	Cross-sectional validation study	Body-size/body-composition assessment in research and clinical settings	Waist, hip, upper-arm and thigh circumferences; body-fat percentage	Test–retest reliability and agreement with reference methods	3D optical body scanner, flexible measuring tape, DXA, digital scale, stadiometer	Evaluation of 3D optical anthropometry for body-size and body-composition monitoring	Home-based 3D optical imaging showed good agreement with manual anthropometry and DXA
7	Moghamis et al. (2020) [21]	Cross-sectional study	Patients undergoing ACL reconstruction	Height, weight, BMI, thigh length, thigh circumference and abdominal girth	Hamstring graft length, graft diameter and tendon thickness	Measuring tape; intraoperative graft measurements	Use of anthropometric characteristics to support ACL graft-size prediction and surgical planning	Height and thigh length were useful predictors of hamstring graft size
8	Hershkovich et al. (2022) [22]	Diagnostic/validation study	Lumbar spine mobility assessment	Schober/modified Schober distance measurements*	Lumbar spinal mobility and CT-derived anatomical measurements	Schober test, modified Schober tests, CT	Validation of clinical spinal mobility measurements against anatomical reference assessment	Modified–Modified Schober testing demonstrated improved accuracy compared with the original method
9	Chandanwale et al. (2024) [23]	Cross-sectional study	Community-dwelling adult women assessed for bone health/osteoporosis	Weight, height, BMI, waist circumference, hip circumference and waist-to-hip ratio	BMD, T-score, BUA, SOS and stiffness index	Digital weighing scale, wall-mounted stadiometer, non-elastic measuring tape, QUS bone densitometer	Evaluation of anthropometric measures as inexpensive indicators of bone health	BMI and body weight were associated with BMD, supporting their potential use as accessible screening indicators of bone health
10	Stribrny et al. (2025) [24]	Cross-sectional study	Individuals with chronic low back pain	Waist circumference and waist-to-height ratio	Respiratory amplitude and diastasis recti	Measuring tape and clinical anthropometric assessment	Evaluation of trunk anthropometric characteristics in relation to chronic low back pain	Greater waist circumference and reduced respiratory amplitude were associated with chronic low back pain
11	Nisanci et al. (2025) [25]	Randomized controlled trial	Individuals with mechanical low back pain	Waist circumference, hip circumference and height	TUG, 40-m fast walk test, balance/mobility outcomes	Stadiometer and measuring tape	Assessment of anthropometric and functional changes following walking rehabilitation	Walking exercise improved mobility, balance and anthropometric measurements compared with the control condition
12	Mohamed et al. (2025) [26]	Diagnostic accuracy study	Adolescents with suspected idiopathic scoliosis undergoing screening	Torso asymmetry, surface depth and trunk morphology	Diagnostic accuracy, sensitivity and specificity	Markerless 3D surface topography and CNN	Digital surface anthropometry for scoliosis screening	Markerless 3D surface topography demonstrated strong diagnostic performance for scoliosis detection
13	Yaşar et al. (2025) [27]	Cross-sectional study	Healthy adults; anthropometry-informed BFR application	BMI, thigh circumference and body-fat percentage	Blood pressure and limb occlusion pressure; regression model	Measuring tape, body-composition analyzer and blood-pressure monitor	Use of anthropometric characteristics to estimate individualized BFR pressure	BMI was reported as the strongest anthropometric predictor of individualized limb occlusion pressure
14	Ghaffari et al. (2025) [28]	Cross-sectional study	Total knee arthroplasty/prosthesis design	Proximal tibial AP length, ML width and tibial morphology	Prosthesis dimensions/fit considerations	Intraoperative photography, AutoCAD software and surgical scale	Anthropometric characterization of the proximal tibia for prosthesis sizing and implant design	Tibial anthropometric characteristics may assist patient-specific prosthesis design and improve implant fit
15	Çıklaçandır and Kaya (2026) [29]	Cross-sectional study	Adults with degenerative scoliosis undergoing gait and balance assessment	BMI; anthropometric body-segment/mass-distribution variables	Center of mass, joint ROM, gait speed, step length and other spatiotemporal gait parameters	Wearable IMU motion-capture suit and anthropometric mass-distribution model	Integration of anthropometric body-segment information with wearable motion analysis in degenerative scoliosis	Wearable motion analysis identified gait asymmetry and balance deficits, supporting objective assessment
16	Cerqueira et al. (2026) [30]	Randomized clinical trial	Patients with knee osteoarthritis undergoing rehabilitation/BFR training	BMI	Limb occlusion pressure, systolic BP and diastolic BP	Doppler ultrasound, pneumatic cuff and sphygmomanometer	Anthropometry-informed characterization of vascular/BFR responses during knee osteoarthritis rehabilitation	Limb occlusion pressure remained relatively stable, suggesting that repeated reassessment may not always be necessary during the intervention
17	Zangi et al., 2026 [31]	Randomized controlled trial	Patients with medial knee osteoarthritis	Weight, height, lower-limb length, knee width, ankle width	Gait and biomechanical outcomes, pain, WOMAC/functional outcomes	Direct anthropometric measurements; caliper for limb/joint widths; 3D motion analysis and force-plate assessment	Anthropometric measurements used for joint-location identification, centre-of-mass estimation, biomechanical modelling, and assessment of knee-brace/local muscle-vibration effects	Individualized anthropometric measurements supported brace fitting; the LMV group showed improvements in pain, WOMAC, knee adduction moment impulse, and knee ROM
18	Kostro et al. (2026) [32]	Randomized controlled trial	Patients with early knee osteoarthritis/gonarthrosis	BMI; foot-alignment variables were anthropometrically defined	Center-of-pressure sway length, COP velocity, ellipse area and KOOS	Force platform/posturography and orthopaedic assessment	Individualized rehabilitation and orthotic intervention in early gonarthrosis	Combined stabilization exercise and custom orthotic intervention improved postural/balance and functional outcomes

Assessment of Risk of Bias

To ensure the credibility of the synthesis data, each selected study underwent a risk-of-bias analysis. The reliability of the provided results was ensured by systematically assessing the relevant literature using validated research methodologies.

The COSMIN Risk of Bias framework was used to assess the methodological quality of studies evaluating the reliability, measurement error, and validity of anthropometric and digital measurement methods. Six measurement-validation studies were assessed using the applicable COSMIN measurement-property domains. Content validity and structural validity were considered not applicable because these studies evaluated anthropometric measurement methods rather than the development or psychometric validation of multi-item measurement instruments. Overall methodological quality was rated as adequate for Bourgeois et al. (2017) and Tinsley et al. (2020), whereas Bragança et al. (2018), Beckmann et al. (2019), Kennedy et al. (2020), and Hershkovich et al. (2022) were rated as doubtful overall. Bourgeois et al. (2017) demonstrated very good methodological quality for reliability and adequate quality for measurement error and criterion validity.

Tinsley et al. (2020) demonstrated very good methodological quality for reliability and measurement error and adequate quality for criterion validity. Bragança et al. (2018) was rated adequate for reliability but doubtful for measurement error and criterion validity. Beckmann et al. (2019) was rated adequate for measurement error but doubtful for criterion validity. Kennedy et al. (2020) was rated adequate for reliability and measurement error but doubtful for criterion validity. Hershkovich et al. (2022) was rated doubtful for measurement error and adequate for criterion validity. In accordance with the COSMIN “worst score counts” principle, the lowest applicable methodological rating determined the overall COSMIN judgement for each study. No conventional low-risk or high-risk classification was assigned to these measurement-validation studies because COSMIN uses the categories very good, adequate, doubtful, and inadequate. Detailed results of the COSMIN risk-of-bias assessment are presented in Table 4.

**Table 4:** COSMIN risk-of-bias assessment of measurement-validation studies COSMIN methodological quality categories: Very good, Adequate, Unclear, and Inadequate. Overall methodological quality for each applicable measurement property was determined according to the COSMIN “worst score counts” principle COSMIN: Consensus-Based Standards for the Selection of Health Measurement Instruments; NA: not applicable

Study	Content validity	Structural validity	Reliability	Measurement error	Criterion validity	Construct validity	Overall COSMIN methodological quality
Bourgeois et al. (2017) [15]	NA	NA	Very good	Adequate	Adequate	NA	Adequate
Bragança et al. (2018) [16]	NA	NA	Adequate	Unclear	Doubtful	NA	Unclear
Beckmann et al. (2019) [17]	NA	NA	NA	Adequate	Doubtful	NA	Unclear
Tinsley et al. (2020) [18]	NA	NA	Very good	Very good	Adequate	NA	Adequate
Kennedy et al. (2020) [20]	NA	NA	Adequate	Adequate	Doubtful	NA	Unclear
Hershkovich et al. (2022) [22]	NA	NA	NA	Unclear	Adequate	NA	Unclear

The risk of bias and applicability of the diagnostic accuracy study were assessed using the QUADAS-2 tool. Mohamed et al. (2025) [26] was judged to have a high risk of bias in the patient-selection domain and the index-test domain, reflecting concerns related to the selection of participants and the conduct and interpretation of the index test. The reference-standard domain was judged to have a low risk of bias, indicating that the reference standard was considered appropriate and its use was unlikely to have introduced substantial bias. The flow and timing domain was judged as having some concerns/unclear risk, because the available information was insufficient to establish with certainty that all participants received the index test and reference standard appropriately and within an adequate time interval.

Regarding applicability, great concern was identified for patient selection, indicating that the study population may not completely represent the population specified in the review question. Low concern was identified for the applicability of the index test and reference standard, suggesting that the index test and reference standard were considered sufficiently relevant to the review question. Overall, Mohamed et al. (2025) [26] was therefore judged to have a high overall risk of bias, primarily driven by concerns regarding patient selection and the index test. These methodological limitations should be considered when interpreting the diagnostic accuracy findings. The complete QUADAS-2 signalling-question responses, domain-level risk-of-bias judgements, and applicability assessments are provided in Supplementary Table 5.

**Table 5:** Risk-of-bias and applicability assessment of the diagnostic accuracy study using QUADAS-2 QUADAS-2: Quality Assessment of Diagnostic Accuracy Studies-2; RoB: risk of bias; AIS: adolescent idiopathic scoliosis; low: low risk of bias/low applicability concern; high: high risk of bias/high applicability concern; unclear: unclear risk of bias due to insufficient reporting

No.	Study	Patient selection – RoB	Index test – RoB	Reference standard – RoB	Flow/timing – RoB	Patient selection – applicability	Index test – applicability	Reference standard – applicability	Overall
1	Mohamed et al. (2025) [26]	High	High	Low	Unclear	High	Low	Low	High risk of bias

The risk of bias of the included randomized controlled trials was assessed using the RoB 2 tool at the level of the specific outcome/result assessed. Four randomized controlled trials were included in the RoB 2 assessment. Nişancı et al. (2025) [25] was judged to have some concerns in the randomization process and selection of the reported result, while the domains relating to deviations from intended interventions, missing outcome data, and measurement of outcomes were judged to have low risk of bias. Consequently, the overall RoB 2 judgement for Nişancı et al. was some concerns. Cerqueira et al. (2026) [30] was judged to have low risk of bias across all five RoB 2 domains for limb occlusion pressure and blood-pressure outcomes and was therefore classified as low risk overall.

Zangi et al. (2026) [31] was judged to have some concerns regarding the randomization process and selection of the reported result, while deviations from intended interventions, missing outcome data, and measurement of outcomes were judged to have low risk of bias; consequently, the overall judgement was some concerns. Kostro et al. (2026) [32] was judged to have low risk of bias in the randomization process, deviations from intended interventions, missing outcome data, and measurement of outcomes, but some concerns regarding selection of the reported result. Therefore, the overall RoB 2 judgement for Kostro et al. was some concerns. Overall, one randomized controlled trial was judged to have low risk of bias, and three were judged to have some concerns. No included trial was judged to have high risk of bias. Detailed RoB 2 domain-level assessments are presented in Table 6.

**Table 6:** Risk-of-bias assessment of included randomized controlled trials using RoB 2 RoB 2: Cochrane Risk of Bias 2; TUG: Timed Up and Go; WOMAC: Western Ontario and McMaster Universities Osteoarthritis Index

S. No.	Study	Outcome/result assessed	Randomization process	Deviations from intended interventions	Missing outcome data	Measurement of the outcome	Selection of the reported result	Overall RoB 2
1	Nişancı et al. (2025) [25]	Anthropometric measurements, TUG, 40-m walk, and balance/mobility outcomes	Some concerns	Low risk	Low risk	Low risk	Some concerns	Some concerns
2	Cerqueira et al. (2026) [30]	Limb occlusion pressure and blood-pressure outcomes	Low risk	Low risk	Low risk	Low risk	Low risk	Low risk
3.	Zangi et al. (2026)[31]	Pain, WOMAC, and biomechanical outcomes	Some concerns	Low risk	Low risk	Low risk	Some concerns	Some concerns
4.	Kostro et al. (2026) [32]	Posturographic and functional outcomes related to personalized training and orthopedic inserts	Low risk	Low risk	Low risk	Low risk	Some concerns	Some concerns

The methodological risk of bias of the analytical cross-sectional studies was assessed using the Joanna Briggs Institute (JBI) Critical Appraisal Checklist for Analytical Cross-Sectional Studies. The eight appraisal items were assessed individually and categorized as Yes, No, or Unclear according to the information reported in each study. The assessment addressed the clarity of the inclusion criteria, description of study participants and setting, validity and reliability of exposure measurement, use of objective and standardized criteria for measurement of the condition, identification and management of confounding factors, strategies for addressing confounding, validity and reliability of outcome measurement, and appropriateness of statistical analysis.

The assessment was reported at the item level, and no overall “high-quality” or “low-risk” classification was assigned to the analytical cross-sectional studies. This approach was adopted because the JBI analytical cross-sectional checklist is used to identify methodological strengths and potential sources of bias across individual appraisal items rather than to generate an unsupported overall quality score.

Across the eight included analytical cross-sectional studies, the criteria for inclusion, participant and setting descriptions, measurement procedures, and statistical analyses were generally adequately reported. However, reporting concerning the identification of potential confounding factors and the strategies used to address confounding varied between studies. Bourgeois et al. (2017) [15], Moghamis et al. (2020) [21], Ghaffari et al. (2025) [28], Stribrny et al. (2025) [24], and Çıklaçandır and Kaya (2026) [29] were judged Unclear for Q5 and Q6 because the available reporting did not provide sufficient information to make a definitive judgement regarding identification and management of confounding factors. The remaining appraisal items for these studies were judged Yes. Chen et al. (2020) [19], Chandanwale et al. (2024) [23], and Yaşar et al. (2025) [27] were judged Yes across all eight appraisal items.

The detailed item-level JBI appraisal is presented in Table 7.

**Table 7:** Item-level critical appraisal of analytical cross-sectional studies using the JBI Critical Appraisal Checklist JBI: Joanna Briggs Institute; Q: appraisal question. Yes: criterion satisfied; no: criterion not satisfied; unclear: insufficient information to make a definitive judgement

S. No.	Author (Year)	Q1. Were the requirements for sample inclusion well-defined?	Q2. Were the study participants and the environment thoroughly described?	Q3. Was the exposure measured in a reliable and valid manner?	Q4. Were the conditions measured using standard, objective criteria?	Q5. Were confounding variables found?	Q6. Were methods for handling confounding variables mentioned?	Q7. Were the results measured in a legitimate and trustworthy manner?	Q8.Was the right statistical analysis applied?
1	Bourgeois et al. (2017) [15]	Yes	Yes	Yes	Yes	Unclear	Unclear	Yes	Yes
2	Chen et al. (2020) [19]	Yes	Yes	Yes	Yes	Yes	Yes	Yes	Yes
3	Moghamis et al. (2020) [21]	Yes	Yes	Yes	Yes	Unclear	Unclear	Yes	Yes
4	Chandanwale et al. (2024) [23]	Yes	Yes	Yes	Yes	Yes	Yes	Yes	Yes
5	Ghaffari et al. (2025) [28]	Yes	Yes	Yes	Yes	Unclear	Unclear	Yes	Yes
6	Yaşar et al. (2025) [27]	Yes	Yes	Yes	Yes	Yes	Yes	Yes	Yes
7	Stribrny et al. (2025) [24]	Yes	Yes	Yes	Yes	Unclear	Unclear	Yes	Yes
8	Çıklaçandır and Kaya (2026) [29]	Yes	Yes	Yes	Yes	Unclear	Unclear	Yes	Yes

Discussion

This systematic review examined the clinical applications of traditional and digital anthropometric measures in orthopaedic assessment and rehabilitation. The included studies demonstrated that anthropometric information is used across several clinical contexts, including musculoskeletal assessment, rehabilitation, diagnostic screening, orthopaedic surgical planning, implant-related assessment, body-composition evaluation, and individualized clinical decision-making. The anthropometric measures identified across the evidence base included body mass index (BMI), body mass, stature, limb and segment dimensions, circumferences, body proportions, trunk morphology, and body-composition characteristics. Importantly, the review distinguished these anthropometric measures from associated clinical and biomechanical outcomes, including joint range of motion, gait parameters, inertial-sensor kinematics, center-of-pressure measures, force-platform outputs, limb occlusion pressure, and blood pressure. This distinction is important because these measures provide complementary clinical information but should not themselves be classified as anthropometric variables.

Clinical Applications of Traditional Anthropometry

The findings indicate that traditional anthropometric measurements remain clinically relevant because they are relatively accessible, inexpensive, and feasible in routine clinical environments. Chen et al. (2020) [19] reported associations between BMI, waist circumference, waist-to-height ratio, inflammation, and disease severity in patients with ankylosing spondylitis. Similarly, Chandanwale et al. (2024) [23] examined anthropometric characteristics in relation to bone-health-related outcomes among community-dwelling women. Collectively, these studies suggest that simple anthropometric characteristics may provide clinically relevant information about body composition, adiposity, and disease-related characteristics.

However, these findings should be interpreted primarily as associational evidence rather than evidence of diagnostic or therapeutic effectiveness. Anthropometric variables such as BMI and waist circumference may be associated with disease severity or other clinical characteristics, but an association does not demonstrate that anthropometric assessment itself changes disease progression or improves patient outcomes. Therefore, anthropometry may be considered a potentially useful component of multidimensional orthopaedic assessment rather than an independent diagnostic or therapeutic intervention.

Anthropometry in Rehabilitation and Functional Assessment

Anthropometric measures were also incorporated into rehabilitation-related assessment. Nişancı et al. (2025) [25] evaluated anthropometric measurements alongside functional outcomes, including the Timed Up and Go test, 40-m fast-walk test, and balance/mobility measures in individuals with mechanical low back pain. Stribrny et al. (2025) [24] investigated waist circumference and waist-to-height ratio in relation to respiratory and trunk characteristics among individuals with chronic low back pain. These findings indicate that anthropometric information can be collected alongside functional assessment and may contribute to characterization of patients undergoing rehabilitation.

Nevertheless, the available evidence does not establish that anthropometric assessment itself improves rehabilitation outcomes. In studies in which anthropometric variables were accompanied by functional outcomes, improvements or associations involving functional outcomes cannot necessarily be attributed to the anthropometric assessment. Anthropometry may provide additional information for patient characterization, monitoring, or treatment planning, but whether incorporating anthropometric assessment into rehabilitation pathways produces superior functional outcomes remains uncertain. This distinction is particularly important because only a limited number of randomized trials directly evaluated rehabilitation-related outcomes, while much of the evidence was observational or measurement-validation research.

Digital and Three-Dimensional Anthropometric Assessment

The review also identified increasing use of digital technologies for anthropometric assessment. Bourgeois et al. (2017) [15], Bragança et al. (2018) [16], Tinsley et al. (2020) [18], and Kennedy et al. (2020) [20] investigated three-dimensional optical or Kinect-based approaches and reported varying levels of agreement, reliability, or measurement validity compared with conventional anthropometric techniques. These findings support the potential of digital approaches to provide standardized and reproducible measurements of body dimensions and morphology.

However, measurement validity should be distinguished from clinical effectiveness. Demonstrating that a digital method agrees with or reliably reproduces a conventional measurement does not establish that its use leads to better diagnosis, treatment selection, rehabilitation adherence, or functional recovery. The principal contribution of these studies is therefore evidence concerning the measurement properties and feasibility of digital anthropometry, rather than evidence of improved patient outcomes.

Mohamed et al. (2025) [26] represents a different application because markerless three-dimensional surface topography combined with convolutional neural network technology was evaluated for scoliosis detection. The diagnostic findings indicate promising performance for digital assessment of trunk morphology and scoliosis classification. Nevertheless, the QUADAS-2 assessment identified important concerns related to patient selection and the index-test methodology. Therefore, the reported diagnostic performance should be interpreted cautiously and should not be generalized directly to routine screening populations without further prospective validation.

Orthopaedic Surgical Planning and Individualized Assessment

Anthropometric variation may also have implications for implant design and prosthesis fit. Ghaffari et al. [28] examined proximal tibial anthropometric characteristics in relation to prosthesis dimensions, highlighting the potential relevance of population-specific anatomical variation to implant sizing. However, the applicability of these findings beyond the population studied should be interpreted cautiously, because anatomical dimensions may vary across populations and healthcare settings. Evidence from a single population does not establish that anthropometry-based implant selection improves postoperative outcomes, and comparative studies across diverse populations are needed to determine whether population-specific anthropometric data translate into clinically meaningful improvements in implant fit, function, or patient-reported outcomes.

Anthropometric measurements may also have a role in orthopaedic surgical planning and individualized treatment. Ghaffari et al. (2025) [28] evaluated proximal tibial anthropometry in relation to prosthesis dimensions and implant considerations in total knee arthroplasty. Moghamis et al. (2020) [21] investigated anthropometric characteristics as predictors of hamstring graft dimensions in patients undergoing anterior cruciate ligament reconstruction. These studies suggest that patient-specific anatomical measurements may assist in preoperative planning, implant selection, or estimation of relevant surgical characteristics.

The clinical importance of these findings lies in the potential to move from population-based assumptions toward patient-specific anatomical assessment. However, evidence that an anthropometric variable predicts an anatomical or surgical characteristic does not necessarily demonstrate improved surgical outcomes. Prospective studies comparing anthropometry-informed treatment planning with conventional planning approaches are therefore needed to establish whether these measurements translate into improved postoperative recovery, implant performance, complication rates, or patient-reported outcomes.

Anthropometry-Informed Rehabilitation and Physiological Applications

Several recent studies examined anthropometric characteristics alongside individualized rehabilitation or physiological interventions. Yaşar et al. (2025) [27] investigated anthropometric predictors of individualized limb occlusion pressure, while Cerqueira et al. (2026) [30] evaluated limb occlusion pressure and blood-pressure responses during blood-flow-restriction training in individuals with knee osteoarthritis. These studies illustrate a potential role for anthropometric information in tailoring physiological or rehabilitation procedures.

Again, the distinction between informing an intervention and improving the outcome of that intervention is important. Anthropometric characteristics may assist in determining individualized parameters or describing patient characteristics, but the current evidence is insufficient to conclude that anthropometry-informed intervention strategies are superior to conventional approaches.

Zangi et al. (2026) [31] further illustrate the potential use of direct anthropometric measurements for brace fitting, joint-location identification, center-of-mass estimation, and biomechanical modelling. The study reported changes in pain, WOMAC, knee adduction moment impulse, and knee range of motion in the intervention groups. These findings are clinically interesting, but the small sample size and limited evidence base restrict the certainty with which they can be generalized. The findings should therefore be considered preliminary evidence supporting further evaluation rather than definitive evidence of effectiveness.

Kostro et al. (2026) [32] examined individualized rehabilitation and orthotic intervention in early gonarthrosis and reported improvements in postural and functional measures. These findings suggest that individualized anatomical or anthropometric information may be incorporated into rehabilitation planning. However, because the available studies differ in interventions, populations, outcomes, and anthropometric variables, their results cannot be combined to establish a general causal effect of anthropometry on rehabilitation outcomes.

Methodological Considerations and Certainty of the Evidence

An important finding of this review is the substantial methodological heterogeneity of the evidence base. The included studies differed in study design, population, clinical condition, anthropometric variables, measurement technology, comparator, and outcome definition. Measurement-validation studies, diagnostic-accuracy studies, observational studies, and randomized controlled trials address fundamentally different research questions and therefore should not be interpreted as providing equivalent levels of evidence.

For this reason, risk of bias was assessed using study-design-appropriate instruments. Measurement-validation studies were assessed using the COSMIN Risk of Bias framework, the diagnostic-accuracy study was assessed using QUADAS-2, randomized controlled trials were assessed using RoB 2 at the specific outcome/result level, and analytical cross-sectional studies were appraised using the JBI analytical cross-sectional checklist. The revised assessment avoids assigning a generic overall quality label to heterogeneous study designs and provides domain- or item-level judgments.

The diagnostic-accuracy evidence should therefore be interpreted separately from measurement-validation evidence, and observational associations should not be interpreted as causal effects. Similarly, the limited number of randomized trials means that evidence concerning whether anthropometry-informed approaches improve rehabilitation outcomes remains insufficient.

Because the available studies were heterogeneous and relatively few studies evaluated the same clinical application and outcome using comparable methods, a pooled estimate across the entire evidence base would not be clinically meaningful. Quantitative synthesis should therefore be restricted to sufficiently homogeneous subsets of studies. Where adequate comparable evidence is available, future updates should report effect estimates with confidence intervals and assess the certainty of evidence using GRADE.

Strengths of the Review

This review has several strengths. First, it considered both conventional and digital anthropometric approaches, thereby reflecting the transition from manual clinical measurement toward three-dimensional and technology-assisted assessment. Second, the review included several orthopaedic and rehabilitation applications, allowing the clinical roles of anthropometry to be considered across assessment, screening, surgical planning, and rehabilitation contexts.

Third, an explicit operational distinction was made between anthropometric measurements and associated clinical, functional, biomechanical, physiological, and motion-analysis outcomes. This reduces the risk of treating gait, joint range of motion, force-platform measures, posturography, inertial-sensor outputs, blood pressure, or limb occlusion pressure as anthropometric measurements.

Fourth, study-design-specific risk-of-bias and critical-appraisal approaches were used rather than applying a single tool across all study designs. The detailed signalling-question assessments provided in the supplementary material further improve transparency and reproducibility.

Finally, the review identifies areas in which evidence is relatively mature, such as measurement validation of selected digital anthropometric technologies, while also identifying areas where clinical effectiveness remains uncertain.

Limitations and Challenges

Several limitations should be considered. First, despite the operational definition used in this review, anthropometry encompasses a broad range of body-size, body-dimension, and body-composition measures, and terminology varied considerably between studies. This heterogeneity made direct comparison difficult. The review therefore deliberately separated anthropometric measurements from associated gait, kinematic, biomechanical, physiological, and functional outcomes.

Second, the review was not prospectively registered in a systematic-review registry. This limits independent verification of whether all methodological decisions were specified before the review was undertaken and should be considered when interpreting the findings.

Third, although multiple major bibliographic databases were searched, the restriction to English-language publications may have introduced language bias. Exclusion of studies for which full text could not be obtained may also have introduced availability bias, because potentially eligible evidence could not be assessed.

Fourth, substantial clinical heterogeneity existed among the included studies. The populations ranged across different orthopaedic and musculoskeletal conditions, while the purposes of anthropometric assessment included disease characterization, screening, measurement validation, surgical planning, individualized intervention, and rehabilitation assessment. In addition, several clinical applications were represented by only one or a small number of studies. This limits the generalizability of findings and prevents reliable conclusions for every clinical application.

Fifth, the evidence base contained relatively few randomized controlled trials directly examining patient-important rehabilitation outcomes. Consequently, the review cannot establish that adding anthropometric assessment to rehabilitation causes improvements in mobility, pain, function, or quality of life.

Sixth, quantitative effect-size synthesis was limited by the small number of sufficiently homogeneous study subsets. Although this limits statistical synthesis, pooling studies with substantially different populations, anthropometric exposures, interventions, and outcomes would potentially produce misleading estimates.

Finally, the certainty of evidence is limited by study-design heterogeneity, small samples in some studies, methodological concerns identified in individual studies, and the limited availability of directly comparable outcome data. Accordingly, the findings should be interpreted as evidence regarding the potential clinical utility and applications of anthropometry, rather than definitive evidence of clinical effectiveness.

Clinical Implications

The findings suggest that anthropometry can serve as a useful component of multidimensional orthopaedic assessment when measurements are standardized, clinically relevant, and interpreted in conjunction with functional and patient-reported outcomes. Traditional measures such as BMI, circumferences, stature, and limb dimensions remain feasible in routine practice, whereas digital approaches may provide additional opportunities for three-dimensional assessment and standardized body-surface characterization.

In clinical practice, anthropometric measurements may be particularly useful for patient characterization, identifying anatomical differences, supporting individualized treatment planning, monitoring selected physical characteristics, and informing certain orthopaedic or rehabilitation procedures. However, anthropometry should be viewed as a complementary assessment approach rather than a replacement for clinical examination, functional assessment, imaging, or validated patient-reported outcomes.

The current evidence does not justify concluding that routine anthropometric assessment independently improves rehabilitation outcomes. Instead, anthropometry may provide information that can contribute to clinical decision-making, and its effectiveness as part of an integrated rehabilitation pathway requires direct evaluation in adequately designed comparative studies.

Future Directions

Future research should prioritize prospective, adequately powered, multicentre studies using standardized anthropometric definitions and measurement protocols. Studies should clearly distinguish anthropometric variables from associated biomechanical, physiological, functional, and motion-analysis outcomes.

Randomized or controlled comparative studies are particularly needed to determine whether incorporating anthropometric assessment into rehabilitation or orthopaedic decision-making produces clinically meaningful improvements in patient-reported outcomes, functional performance, treatment adherence, postoperative recovery, or quality of life. For digital anthropometry, future studies should also evaluate external validation, inter-rater and test-retest reliability, measurement error, generalizability across populations, and performance under routine clinical conditions.

Where multiple studies evaluate comparable populations and outcomes, future research should report standardized effect estimates and confidence intervals and undertake meta-analysis when clinically and methodologically appropriate. Certainty of evidence should also be evaluated using GRADE where sufficient evidence exists.

Finally, integration of three-dimensional imaging, artificial intelligence, and digital anthropometry may expand the ability to characterize patient-specific anatomy and morphology. However, technological accuracy should not be equated with clinical effectiveness; future research should demonstrate whether these technologies lead to better clinical decisions and meaningful improvements in patient outcomes.

## Conclusions

Traditional and digital anthropometric measures may have applications in selected orthopaedic and rehabilitation settings, including body-composition assessment, clinical characterization, screening, surgical planning, and clinical monitoring. Their validity and clinical usefulness vary with the measurement method, assessment protocol, population, and intended purpose. Current evidence supports the validity, reliability, or potential clinical application of selected anthropometric approaches in specific contexts; however, it does not establish that routine anthropometric assessment improves clinical decision-making, recovery, functional outcomes, or rehabilitation effectiveness. Findings from measurement-validation, observational, and cross-sectional studies should not be taken as evidence that anthropometric assessment itself improves patient outcomes. The considerable heterogeneity in study designs, populations, anthropometric measures, technologies, and outcome variables restricted direct comparison and quantitative synthesis. Future studies should be prospective and adequately powered, and should use standardized anthropometric protocols, design-appropriate methodological assessment, external validation of digital technologies, and patient-centered outcomes to determine whether incorporating anthropometry into routine orthopaedic and rehabilitation practice provides meaningful clinical benefit.
